# SEPT12-Microtubule Complexes Are Required for Sperm Head and Tail Formation

**DOI:** 10.3390/ijms141122102

**Published:** 2013-11-07

**Authors:** Pao-Lin Kuo, Han-Sun Chiang, Ya-Yun Wang, Yung-Che Kuo, Mei-Feng Chen, I-Shing Yu, Yen-Ni Teng, Shu-Wha Lin, Ying-Hung Lin

**Affiliations:** 1 Department of Obstetrics & Gynaecology, National Cheng Kung University, No. 1, University Road, Tainan City 701, Taiwan; E-Mails: paolink@mail.ncku.edu.tw (P.-L.K.); vic0009@yahoo.com.tw (Y.-Y.W.); 2 Graduate Institute of Basic Medicine, Fu Jen Catholic University, No. 510, Zhongzheng Road, Xinzhuang District, New Taipei City 242, Taiwan; E-Mail: 053824@mail.fju.edu.tw; 3 Graduate Institute of Basic Medical Sciences, National Cheng Kung University, No. 1, University Road, Tainan City 701, Taiwan; E-Mail: s5895144@mail.ncku.edu.tw; 4 Research Center for Emerging Viral Infections, Chang Gung University, No. 259 Wen-Hwa 1st Road, Kwei-Shan Taoyuan 333, Taiwan; E-Mail: b583202@ms3.hinet.net; 5 Laboratory Animal Center, National Taiwan University College of Medicine, No. 1, Sec. 4, Roosevelt Road, Taipei 106, Taiwan; E-Mail: oxfo@seed.net.tw; 6 Department of Biological Sciences and Technology, National University of Tainan, No. 33, Sec. 2, Shulin Street, West Central District, Tainan City 700, Taiwan; E-Mail: tengyenni1968@gmail.com; 7 Clinical Laboratory Sciences and Medical Biotechnology, National Taiwan University, National Taiwan University Hospital, No. 1, Sec. 4, Roosevelt Road, Taipei 106, Taiwan; E-Mail: mtshuwha@ntu.edu.tw

**Keywords:** spermiogenesis, SEPT12, microtubules

## Abstract

The septin gene belongs to a highly conserved family of polymerizing GTP-binding cytoskeletal proteins. SEPTs perform cytoskeletal remodeling, cell polarity, mitosis, and vesicle trafficking by interacting with various cytoskeletons. Our previous studies have indicated that *SEPTIN12^+/+/+^*^/−^ chimeras with a *SEPTIN12* mutant allele were infertile. Spermatozoa from the vas deferens of chimeric mice indicated an abnormal sperm morphology, decreased sperm count, and immotile sperm. Mutations and genetic variants of *SEPTIN12* in infertility cases also caused oligozoospermia and teratozoospermia. We suggest that a loss of SEPT12 affects the biological function of microtublin functions and causes spermiogenesis defects. In the cell model, SEPT12 interacts with α- and β-tubulins by co-immunoprecipitation (co-IP). To determine the precise localization and interactions between SEPT12 and α- and β-tubulins *in vivo*, we created *SEPTIN12*-transgene mice. We demonstrate how SEPT12 interacts and co-localizes with α- and β-tubulins during spermiogenesis in these mice. By using shRNA, the loss of *SEPT12* transcripts disrupts α- and β-tubulin organization. In addition, losing or decreasing SEPT12 disturbs the morphogenesis of sperm heads and the elongation of sperm tails, the steps of which are coordinated and constructed by α- and β-tubulins, in *SEPTIN12^+/+/+^*^/−^ chimeras. In this study, we discovered that the *SEPTIN*12-microtubule complexes are critical for sperm formation during spermiogenesis.

## 1. Introduction

### 1.1. Septins

Septins are a highly conserved polymerizing GTP-binding protein family. The functions of SEPTs are diverse, and include establishing cell polarity, cytoskeletal remodeling, membrane compartmentalization, cell cycle progression, and vesicle trafficking. Among the 14 members of the septin gene family in mammalian species, several members are expressed ubiquitously, whereas others are expressed only in well-differentiated cells (e.g., neurons or male germ cells) [1]. SEPT proteins form homomeric or heteromeric filament-like structures in cells, such as the SEPT2/6/7 complex. The loss of one component in a complex may cause the levels of other septins to decrease [2,3]. Recent studies have demonstrated the vital role of septins in various pathological processes, including Alzheimer’s disease, hereditary neuralgic amyotrophy, leukemia, ovarian tumors, breast cancer, and male infertility [4,5].

### 1.2. SEPT Interaction with Cytoskeletons

SEPTs play a crucial role in cytokinesis by interacting with other cytoskeletons. In yeast, septin polymerization mainly occurs at the cortical sites of the mother-bud neck by coordinating with the actomyosin contractile ring [6]. In the mammalian species, SEPTs interact with several cytoskeletal proteins (e.g., actin, myosin II, and tubulins). For example, Kinoshita *et al*. indicated that SEPT2 interacts with actin-based structures in NIH-3T3 cells [7]. The disruption of actin structures subsequently affects the formation of SEPT2 and SEPT4 filamentous patterns in the same cell line [2,7,8]. SEPT2 also co-localizes and associates with microtubulins in PC12 cells [9]. For example, SEPT2 binds directly to the non-muscle myosin II, and the disruption of the SEPT2-myosin II interaction results in the loss of stress fibers in interphase cells, causing the ingressed cleavage furrow in dividing cells to become unstable [10]. During cell cycle progression, SEPT2 interacts with centromere-associated protein E (CENP-E), a microtubule motor protein, to stabilize the binding between kinetochore and spindle microtubules [11,12]. The loss of SEPT2 results in chromosome loss from the metaphase plate, a lack of chromosome segregation and spindle elongation, and incomplete cytokinesis. Interrupting tubulins by nocodazole, an antimitotic agent, also alters SEPT2 localization. Kremer *et al*. demonstrated that microtubulin stability is regulated by the SEPT2/6/7 complex through an interaction with microtubule-associated protein 4 (MAP4) [3]. SEPT9 also interacts with microtubulins during the interphase and co-localizes with mitotic spindles through microtubulins during mitosis in various cell lines [13,14]. In the rodent reproductive system, SEPT2/7/9, β-tubulins, and other proteins form a stable intercellular bridge between mother and daughter cells during syncytium formation [15].

### 1.3. SEPT12 and Other SEPTs in Spermatogenesis

Recent studies have revealed the pivotal roles of SEPTs in mammalian spermatogenesis [16–18]. SEPT4 is located at the annulus, a ring-like structure connecting the mid-piece and the principal piece of the flagellum, and is crucial for maintaining the proper mitochondrial architecture of the mid-piece and the integrity of the annulus in the sperm tail [16,17]. *Septin4^−/−^* mice were viable, but the males were sterile and possessed immotile sperm accompanied by a defective annulus. In humans, septins (SEPT1/4/6/7) were lost in most of the spermatozoa in asthenozoospermia patients [16,19,20]. We identified *SEPTIN12* as a potential sterile gene by conducting a cDNA microarray analysis on the testicular tissue [21] and determined that SEPT12 was expressed in post-meiotic male germ cells [22]. We also generated 129 embryonic stem cells with a *SEPTIN12* mutant allele deleted in the exons encoding the *N*-terminal GTP-binding domain. Most chimeras derived from the targeted ES cells were infertile, and the few fertile chimeras produced only offspring with the C57 BL/6 wild-type (WT) background. The residual spermatozoa isolated from the vas deferens exhibited distinctive morphological defects—a round head, a broken acrosome, and a bent tail [23]. In humans, *SEPTIN12* mutations and genetic variants were identified in infertile men presenting oligoasthenozoospermia with distinctive sperm pathology: a bent tail and a defective annulus [24,25]. We also observed a round head and a broken acrosome in the spermatozoa of infertile men with SEPTIN12 mutations and genetic variants (unpublished data).

### 1.4. Roles of α- and β-Tubulins during Spermatogenesis

During spermatogenesis, microtubules are expressed at various developmental stages [26–28]. During spermatogenesis, microtubule filaments are dispersed as networks without any obvious centrosome in the pachytene spermatocytes. During spermiogenesis, microtubule filaments are enriched around the sperm nucleus in step 7 spermatids. In step 8–15 spermatids, microtubule expression increases to facilitate manchette formation, a transient microtubule and actin-containing structure that is vital for nuclear and head shaping and tail formation [29–33]. As spermiogenesis advances, α- and β-tubulins elongate from the centrosome to form the sperm tail and are discreetly arranged into a unique 9 + 2 structure with other components of the flagella axoneme.

Previous studies have suggested that microtubule dynamics are critical for sperm head and tail formation during mammalian spermiogenesis [30,31,33]. We discovered that a reduced SETP12 expression or SEPT12 dysfunction is associated with extensive sperm pathologies, including a round head and a bent tail. Because SEPTs are involved in numerous cellular processes through microtubule regulation, we hypothesized that SEPT12 deficiency may disrupt microtubule dynamics during spermiogenesis. We demonstrate that SEPT12 forms α- and β-tubulin complexes during spermiogenesis, and that SEPT12 deficiency disrupts α- and β-tubulin dynamics *in vivo* and *in vitro*. Our findings suggest that the SEPT12-microtubule complex is involved in forming the sperm head and tail.

## 2. Results and Discussion

### 2.1. SEPT12 Interacts with α- and β-Tubulins in the Male Germ Cell Line

**Figure 1:**
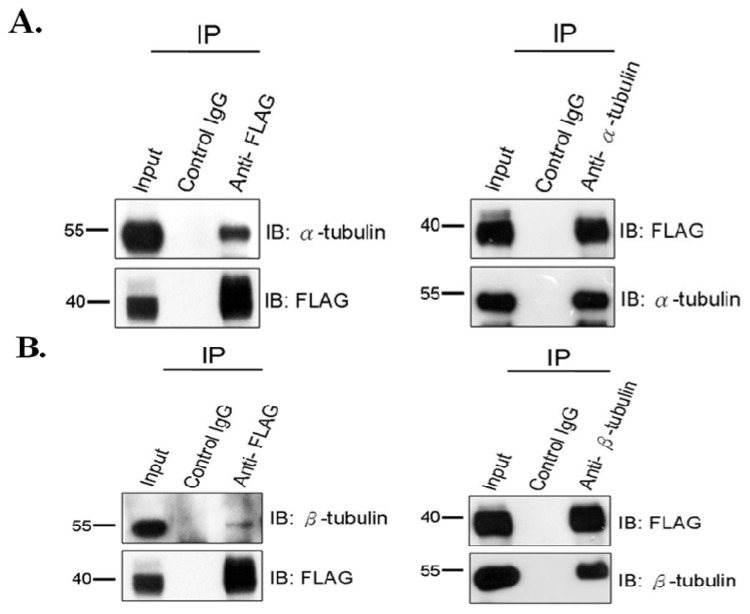
SEPT12 interacts with α-tubulin and β-tubulin in male germ cell line. (**A**) Co-IP of FLAG-SEPT12 and α-tubulin. Lysates from transfected NTERA-2 d.D1 cells were immunoprecipitated (IP) with anti-FLAG antibody (**left** panel, lane3), anti-α-tubulin (**right** panel, lane 3), or a nonspecific control IgG (**left** and **right** panel, lane 2), followed by immuno-blotting (IB) with anti-α-tubulin or anti-FLAG antibody. Input protein (5%) was used as control of immuno-blotting (IB) in the transfected cell lysates; (**B**) Co-IP of FLAG-SEPT12 and β-tubulin. The data presents as (**A**).

Previous studies have indicated that SEPTs interact with α- and β-tubulins and participate in cytokinesis occurring in somatic cells and the neurite outgrowth of PC12 [3,9,13,14]. Considering that α- and β-tubulins become major components of the sperm head and sperm tail during spermiogenesis, we applied the co-immunoprecipitation (co-IP) assay to test whether SEPT12 interacts with α- and β-tubulins in NTERA-2 pluripotent human testicular embryonal carcinoma (d.D1) cells. The NTERA-2 d.D1 cells were transfected using SEPT12-FLAG, followed by immunoprecipitation (IP) using anti-FLAG, the anti-α-tubulin, and the anti-β-tubulin antibody. The co-IP experiments demonstrated that the α-tubulin was pulled down with SEPT12-FLAG, which was recognized by the anti-α-tubulin antibody (Figure 1A, left). Conversely, SEPT12-FLAG also pulled down with α-tubulin (Figure 1A, right). The co-IP experiment produced the same result for β-tubulins (Figure 1B) and in 293T cells (Figure S1). Thus, SEPT12 interacts with α- and β-tubulins in the cell model.

### 2.2. SEPT12, α- and β-Tubulins form the Complex *In Vivo*

**Figure 2:**
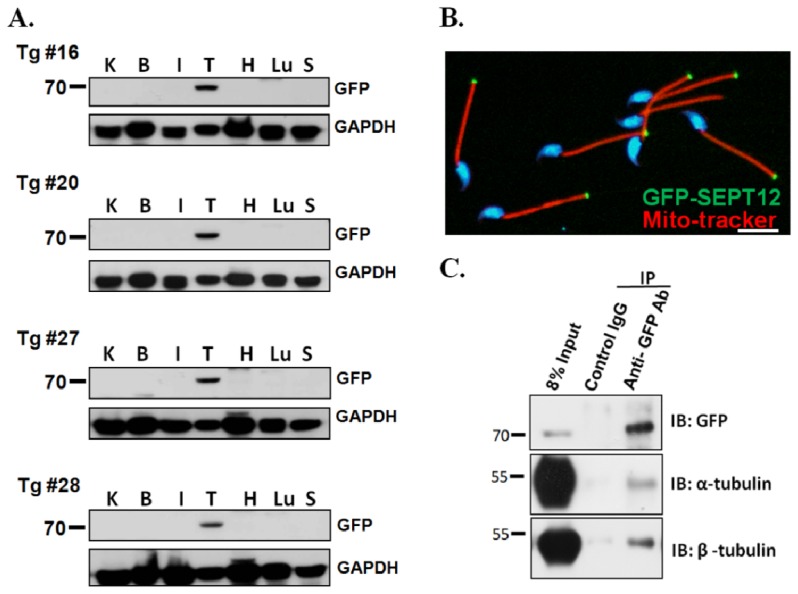
Expression of SEPT12, α- and β-tubulin in the *SEPTIN12*-transgenic mice. (**A**) SEPT12 expression in different organs from the *SEPTIN12*-transgenic mice. The tissue lysate from kidney (K), brain (B), intestine (I), testis (T), heart (H), lung (L) and spleen (S) were analyzed by Western blotting with anti-GFP or anti-GAPDH antibody (loading control); (**B**) Spermatozoa isolated from vas deferens of *SEPTIN12*-transgenic mice were stained with mito-tracker (Red), DAPI (Blue) and SEPT12 (Green); Scale bar: 5 μm; (**C**) Co-IP of GFP-SEPT12 and α- or β-tubulin. Testicular lysates from *SEPTIN12*-transgenic mice were immunoprecipitated (IP) with anti-GFP antibody (lane3) followed by immuno-blotting (IB) with anti-GFP, anti-α-tubulin and anti-β-tubulin antibody, respectively. IgG was used as control. Input protein (8%) was used as control of immunoblotting (IB) in the testicular lysates.

To demonstrate the interaction and precise localization and interactions between SEPT12 and α- and β-tubulins complexes *in vivo*, *SEPTIN12*-transgenic mice were developed by overexpressing a plasmid containing a full-length *SEPTIN12* sequence tagged with GFP. The plasmid was driven by the endogenous *SEPTIN12* promoter, which spans 2000 bp from the translational start codon. More than 25 *SEPTIN12*-trangenic mice were created. The *SEPTIN12* copy number in transgenic mice was evaluated using real-time PCR (Figure S2). The testis-specific expression of SEPT12-GFP was discovered in these transgenic mice (Figure 2A), and was similar to that of endogenous SEPT12 [22]. We also determined that SEPT12-GFP was located at the sperm annulus in the spermatozoa isolated from the vas defense of the transgenic mice (Figure 2B). We used the testicular tissues from the *SEPTIN12-*transgenic mice for the co-IP experiments. Figure 2C shows that the α- and β-tubulins were pulled with an anti-GFP antibody. These findings indicate that SEPT12 interacts with α- and β-tubulins *in vivo*.

### 2.3. Co-Localisation of SEPT12 with α-/β-Tubulins *in Vivo*

**Figure 3:**
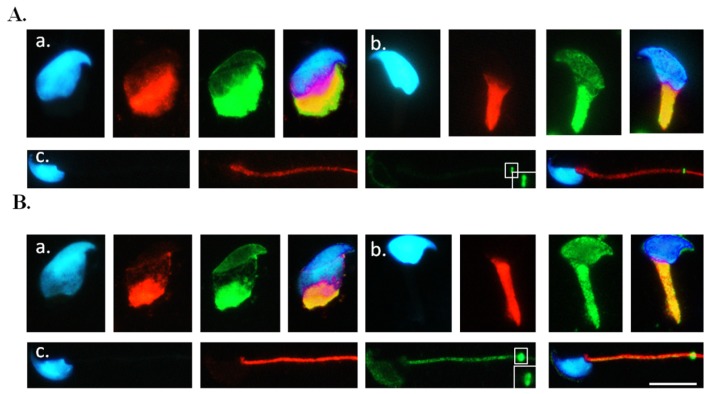
SEPT12 is co-localized with α- and β-tubulin at different steps of spermiogenesis in the *SEPTIN12*-transgenic mice. Immuno-fluorescence detection of SEPT12 and α-tubulin (**A**) or β-tubulin (**B**). Anti-EGFP antibody (**A** and **B**; Green), anti-α-tubulin (**A**, Red), anti-β-tubulin (**B**, Red) and DAPI (**A** and **B**; Blue). (**a**) early elongation stage; (**b**) elongating; (**c**) mature sperm. From left to right: DAPI (Blue), tubulin (Red), SEPT12 (Green) and merge of pictures. Enlarge figures are shown in right lower corner of (**c**). Scale bar : 5 μm.

The α- and β-tubulins have been used as sperm-head markers in elongating spermatids [29,30,34–36]. To determine whether SEPT12 co-localizes with α- and β-tubulins in elongating and mature spermatozoa *in vivo*, we isolated the various developmental stages of the spermatid derived from the *SEPTIN12*-transgenic mice, and then co-stained the spermatid with anti-GFP and anti-α-tubulin or anti-β-tubulin antibodies. The immunofluorescence assay (IFA) revealed that certain SEPT12-GFP were co-localized with α- and β-tubulins when the sperm head was shaped (Figure 3Aa,Ba). In the elongating spermatids, SEPT12-GFP and α- and β-tubulins were co-localized in the neck and tail regions (Figure 3Ab,Bb). Finally, in the mature sperm, SEPT12 signals localized at the annulus on the sperm tail, consisting primarily of α- and β-tubulins (Figure 3Ac,Bc). The co-localization of endogenous SEPT12 and α- and β-tubulins was also observed in the spermatids of the wild-type mice (Figure S3). These findings suggest that SEPT12-α- and SEPT12-β-tubulin complexes are involved in sperm head and tail formation during spermiogenesis.

### 2.4 *. SEPTIN12* Knockdown Disrupts the α- and β-Tubulin Patterns and Reshapes Cell Morphology

**Figure 4:**
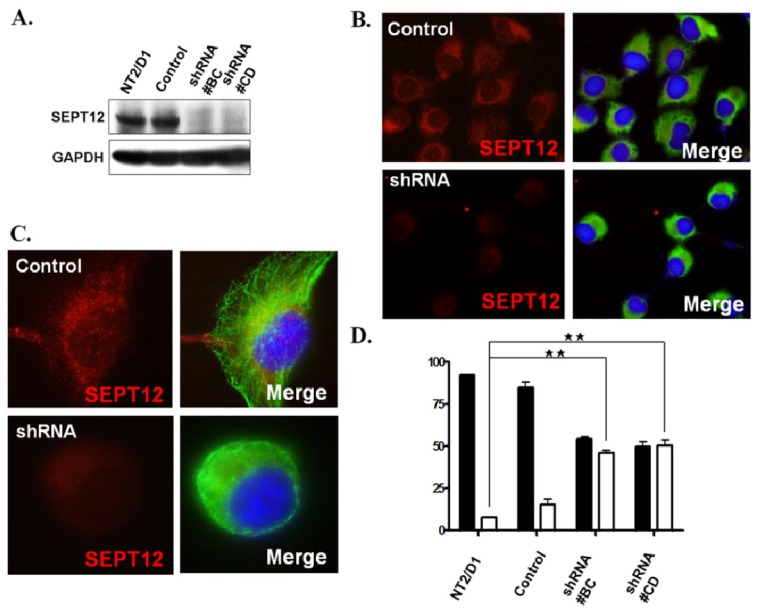
Silencing *SEPTIN12* affects α-/β-tubulins organization in the NTERA-2 d.D1 cell. (**A**) Expression levels of SEPT12 in the stable clone derived from the NTERA-2 d.D1 cell (shRNA #BC and #CD for *SEPTIN12*). Control: a shRNA for the GFP sequence. Each lane was probed with the antibody against SEPT12 or GAPDH (as a loading control) in immuno-blotting; (**B**,**C**) Immuno-fluorescent detection of SEPT12 (Red) and α-tubulin (Green) in NTERA-2 d.D1 cells treated with shRNA for GFP (control) or *SEPTIN12*. Cells were stained with anti-SEPT12 (red, **left** panel) and merge of images for anti-SEPT12 and anti-α-tubulin (green, **left** panel). Magnification ×400 (**B**) and ×1000 (**C**); (**D**) Quantification of disorganized α-tubulin in the *SEPTIN12* knockdown cell. There are two distinctive patters of α-tubulin in the *SEPTIN12* knockdown cell: the polymerized form (black bar) and the disorganized form (white bar). The percentages of two distinct patterns were calculated by randomly selected cells (*n =* 300, duplication). Two-tailed Student’s *t-*test; Error bars indicate ± SEM (**, *p*< 0.001).

Because the co-IP and IFA results suggest that SEPT12-α- and SEPT12-β-tubulin complexes may be involved in sperm head and tail formation, we investigated whether a low dosage of SEPT12 may affect the α- and β-tubulin structure and cell shape in cells. Figure 4A displays the efficient knockdown of *SEPTIN12* transcripts in the shRNA#BC and #CD clones of the NTERA-2 d.D1 cell lines by RNA interference. SEPT12-depleted cells were doubly stained with anti-SEPT12 and the anti-α-tubulin antibody to probe the expression patterns of SEPT12 and α-tubulins. Compared to cells that were not SEPT12 depleted, the *SEPTIN12*-depleted cells lost the SEPT12 signal dramatically and presented distinct α-tubulin structures and cell morphology (Figure 4B). In the control cells, α-tubulins formed a highly organized structure by polymerization around the cytoplasm, termed “a polymerized form.” Conversely, in SEPT12-depleted cells, α-tubulin was not polymerized (termed “a disorganized form”) and the cell shape was altered (Figure 4C). A similar result was also observed in the experiment using β-tubulins (data not shown). Fewer than 20% of the cells with disorganized microtubules existed in untransfected wild-type and scramble-transfected cells. The percentage of cells with disorganized α- and β-tubulins increased to 50% in the SEPT12-depleted cell lines (Figure 4D). These findings suggest that SEPT12 is involved in regulating α- and β-tubulin organization *in vitro* and that a reduced amount of SEPT12 affects α- and β-tubulin polymerization and cell morphology.

### 2.5. Reduced SEPT12 Disrupts Sperm Head and Tail Formation in *SEPTIN12*^+/+/+/−^ Mice

**Figure 5:**
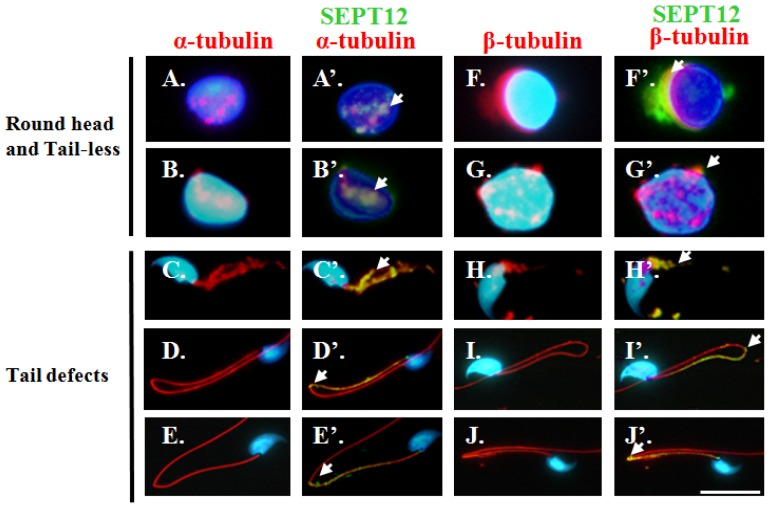
Reduced SEPT12 expression influences the α- and β-tubulins structure of head and tail in spermatozoa isolated from the *Septin12*^+/+/+/−^ chimeras. Analysis of spermatozoa from *Septin12*^+/+/+/−^chimeras mice (**A**–**J**′). Spermatozoa were stained with anti-α-tubulin (**A**–**A**′, **B**–**B**′, **C**–**C**′, **D**–**D**′, and **E**–**E**′; red signal), anti-β-tubulin (**F**–**F**′, **G**–**G**′, **H**–**H**′, **I**–**I**′, and **J**–**J**′; red signal), and co-staining with anti-SEPT12 antibody (**A**′–**J**′). Arrow indicates SEPT12 signals in spermatozoa. Scale bar: 5 μm.

We previously discovered that an excessive amount of sperm from the vas deferens of *SEPTIN12^+/+/^*^+/−^ chimeric mice possessed round heads and no tails (wild type: 0%, *n* = 14; *SEPTIN12*^+/+/+/−^: 60.8% ± 40.3%, *n* = 15; *p* < 0.05), and also possessed sperm with tail defects (wild type: 10.9% ± 3.6%; *n* = 14; *SEPTIN12*^+/+/+/−^: 37.5% ± 7.9%, *n* = 9; *p* < 0.05) [22]. To determine whether reduced SEPT12 affects the α- and β-tubulin structure of the sperm head and tail structure and subsequently causes sperm defects *in vivo*, we performed IFA on these spermatozoa. One group of sperm (round head and tailless) from the vas deferens of the *SEPTIN12*^+/+/+/−^ chimeric mice exhibited disorganized or split α- and β-tubulin signals surrounding the nucleus and the sperm head was not formed adequately (Figure 5A,B,F,G). The α- and β-tubulin remained partially co-localized with SEPT12 (Figure 5A′,B′,F′,G′). The other sperm exhibited disorganized (Figure 5C,H) or bent tails (Figure 5D,E,I,J), which are composed of α- and β-tubulin. SEPT12 signals were scattered around the mid-piece, neck, annulus, and principal piece (Figure 5C′,D′,E′,H′,I′,J′). Conversely, sperm with wild-SEPT12 displayed a highly organised pattern of α- and β-tubulins (Figure 3 and Figure S3) compared with sperm from the chimeric mice. These data indicated that a reduced SEPT12 level causes disorganized sperm head formation and disrupted tail elongation, the steps of which are coordinated and constructed using α- and β-tubulins, in certain male germ cells.

## 3. Experimental Section

### 3.1. Cloning and Transfection

The full lengths of *SEPTIN12* were RT-PCR-amplified from a human RNA panel and cloned into the pFLAG-CMV2 or pEGFP-N1 vector, as previously described [25]. All of the constructs were confirmed by using DNA sequencing. After transfecting the plasmids in the cell line by using Lipofectamine 2000 (Invitrogen, Carlsbad, CA, USA), the cells were subjected to Western blot analysis, IFA, or a co-IP assay.

### 3.2. Co-Immunoprecipitation Assay

The NTERA-2 d.D1 (NT2D1) was used for the co-IP experiment. The co-IP analysis was performed according to the procedures in our previous study [37]. The pFLAG-*SEPTIN12* plasmids were transfected into cells using Lipofectamine 2000 (Invitrogen, Carlsbad, CA, USA). The cell lysates containing 4 mg of protein in 1 mL were pre-cleared by incubating with 50 μL of protein A/G beads (Santa Cruz Biotechnology, Santa Cruz, CA, USA) for 1 h at 4 °C on a rotator. The clear supernatant was incubated overnight with the control IgG, the monoclonal anti-FLAG (Sigma-Aldrich, St. Louis, MO, USA, F-1804), the monoclonal anti-α-tubulin antibody (Sigma-Aldrich, St. Louis, MO, USA, T 9026), the monoclonal anti-β-tubulin antibody (Sigma-Aldrich, St. Louis, MO, USA, T 4062), or anti-GFP (Santa Cruz Biotechnology, Santa Cruz, CA, USA. sc-9996). The samples were then washed twice with 1× PBS followed by Western blotting. The following antibodies were used in the Western blot analysis: anti-tubulins, anti-FLAG, and anti-SEPT12 antibodies (Abnova, Taipei, Taiwan, H00124404- B01P). Western blot analysis was performed according to standard protocol [24].

### 3.3. Creation of *SEPTIN12*-Transgenic Mice

The promoter and the full-length cDNA of the *SEPTIN12* gene were constructed into pFLAG-CMV2 plasmids. The core promoter region of *SEPTIN12* has been defined in previous studies (unpublished data). The size of the core promoter was approximately 2 kb and was located in the region upstream of Exon 1, encompassing Exon 1 and Exon 2. Green fluorescent protein (GFP) tags were constructed into this vector. Intracytoplasmic sperm injection (ICSI) was used to inject the plasmid clones into the fertilized oocytes (pronuclear-stage embryos) to create transgenic mice. The copy number *SEPTIN12*-GFP was evaluated using real-time PCR, as previously described [37].

### 3.4. Separation of Murine Testicular Germ Cell Populations and Sperm Preparation

We separated the spermatogenic cells based on the density of the cell types by using a centrifugal system previously described [37]. The animal studies were all approved by the Animal Care Review Board of National Cheng-Kung University Medical College. After decapsulation and enzyme digestion, germ cell suspensions were filtered through 35 μM nylon filters (Falcon, Becton Dickinson, Franklin Lakes, NJ, USA), and then centrifuged using a Kubota centrifuge 3330 (Kubota Corp, Tokyo, Japan). Germ cells in various developmental stages were collected. Mature spermatozoa were collected from the vas deferens of adult male mice. Finally, the suspensions were centrifuged using maximal force for 10 min, and were then spread onto a slide and air dried.

### 3.5. Immunofluorescence Assay

In the IFA, the slides from the transfected cells or male germ cells isolated from the testicular tissues of mice were treated with 0.1% Triton X-100 and washed twice with Tris-buffered saline (TBS), and then incubated with the primary antibody for 60 min at room temperature. After washing with TBS, the sections were exposed to either the Alexa Fluor^®^ 488 goat anti-mouse IgG antibody (Invitrogen, Carlsbad, CA, USA, cat no. A-11001) or the Alexa Fluor^®^ 568 goat anti-rabbit IgG antibody (Invitrogen, Carlsbad, CA, USA, cat no. A-11008) for 60 min at room temperature, and then washed again with TBS. Mitotracker Red CMXRos (Invitrogen, Carlsbad, CA, USA, M7510) and 4′,6-diamidino-2-phenylindole (DAPI) (Invitrogen, Carlsbad, CA, USA, D3571) were used to stain the mitochondria and nuclei, respectively.

### 3.6. Creation of a Stable Clone by Transfecting with SEPTIN12 shRNA

The shRNA clones were obtained from the National RNAi Core Facility located at the Institute of Molecular Biology/Genomic Research Centre, Academia Sinica, supported by the National Core Facility Program for Biotechnology Grants of NSC 100-2319-B-001-002. Two clones (TRCN0000155625 and TRCN0000156154) were used to generate a stable assay. After transfecting the NT2D1 cell line, purmycin was used to select stable clones, which were then used for Western blotting and IFA. Western blot analysis was performed using the anti-SEPT12 antibody (Abnova, Taipei, Taiwan, H00124404-B01P) according to standard protocol [24].

## 4. Conclusions

We found that the co-localization and interaction of SEPT12 with α- and β-tubulins are major components in the development of sperm heads and tails. We also determined that a reduced SEPT12 expression affects α- and β-tubulin organization in cells and produces defective sperm heads and tails in *SEPTIN12^+/+/+/−^* chimeric mice. Based on these findings, we proposed that the SEPT12-α- and SEPT12-β-tubulin complexes play a pivotal role in spermiogenesis.

### 4.1. Coordination between SEPTs and Microtubules

SEPTs are cytoskeletal scaffold elements. A growing consensus indicates that they may play a broader role in microtubule-dependent processes, such as cellular morphogenesis, cell cycle progression, cell motility, exocytosis, and cell-shape maintenance by recruiting and interacting with other proteins [4,38,39]. Previous studies have indicated that microtubule disruption disturbs SEPT organization [13,40]. For example, SEPT9 is localized with microtubules, and treating it with nocodazole, a specific inhibitor of microtubular polymerization, causes SEPT9 filament disruption [13]. By contrast, treating HMEC cells with cytochalasin B, a specific inhibitor of actin polymerization, does not affect the filament distribution of SEPT9 [14]. Furthermore, Kremer *et al*. identified MAP4 as a septin partner for regulating microtubule stability [3]. In this study, we discovered a crucial dynamic interaction between SEPT12 and α- and β-tubulins for sperm head and tail formation during spermiogenesis.

### 4.2. SEPT12 Depletion May Affect Cytoskeleton Organization

Several studies have recently demonstrated that SEPT depletion disrupts the cell shape by affecting the binding of the cell with microtubules or actins [3,41–43]. SEPT2 fibers co-localize with modified microtubules (polyGlu tubulin), and deplete the SEPT2 expression by siRNAs, which results in a reduced expression of polyGlu microtubules and a loss of the columnar shape of epithelial cells. This finding indicates that SEPT2 is essential for preserving polyGlu microtubules, which are involved in maintaining cellular morphology [41]. SEPT7 knockdown also produces a stabilized cell morphology by increasing the level of acetylated tubulins, a marker of stabilized microtubules, even after treatment with nocodazole [3]. In this study, reduced SEPT12 in NTERA-2 d.D1 cells disrupted α- and β-tubulin organization and influenced the cell morphology. Reduced SETP12 likely disrupts sperm head and tail formation by affecting the α- and β-tubulins.

### 4.3. Sperm Head and Tail Formation during Spermiogenesis

**Figure 6:**
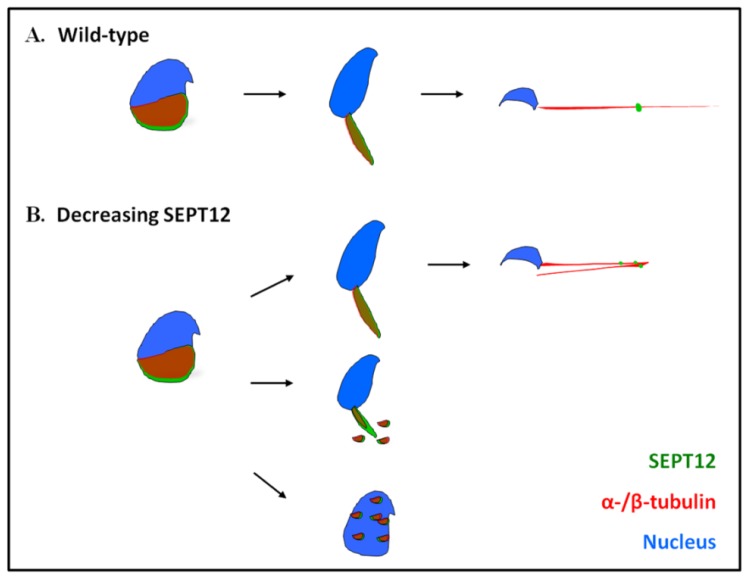
A working model of SEPT12 and α-/β-tubulins during spermiogenesis. (**A**) In the wild-type spermatozoa, SEPT12 forms the manchette structure of head, which is constituted with α-/β-tubulins. During sperm elongation, both SEPT12 and α-/β-tubulins are concentrated at the elongated midpiece to assure its structural integrity and facilitate tail formation. α-/β-tubulins are the major components of the axoneme of the mature sperm tail; (**B**) By reducing the SEPT12 level, the organization of α-/β-tubulins is disrupted. For spermatoza that are capable of completing tail elongation, they are presented with defective annulus with tail folded at 180 degrees (**Upper** panel). For spermatzoa with severe dysfunction of SEPT12 and α-/β-tubulins complex, disturbed structure of tail (**middle**) or round head with less tail. Red: α-/β-tubulins; green: SEPT12; Blue; nucleus.

During spermiogenesis, post-meiotic male germ cells undergo drastic morphological changes, accompanied by the formation of a transient manchette, which is organized by α- and β-tubulins for sperm head shaping [29–33]. The manchette is an important structure formed in step 8 spermatids and disappears in step 15 spermatids. The manchette elongates and condenses the spermatid nucleus and grows the centrosome-divided axoneme [29,31,32]. Several genes are involved in manchette formation. Among these, *Hook1* is critical for manchette and tail formation [29,36]. Deleting Exons 10 and 11 of the *Hook1* gene produces a non-functional protein and subsequently creates an “abnormal spermatozoon head shape” (azh) murine strain. Hook1 is expressed at the manchette and from the attachment site of the flagellum to the nucleus in the elongated spermatids. Hook1 interacts directly with the manchette through the microtubule-binding domain localized in the *N*-terminal region of the protein [44]. Hook1 deletion disrupts the manchette-microtubule complexes and causes abnormalities of the manchette, sperm head shaping, and sperm tail integrity in the Azh strain [29,30,45]. In this study, we discovered that SEPT12 interacts with α- and β-tubulins, and decreased *SEPTIN12* causes disorganized sperm head formation and defective tail elongation in certain spermatozoa. Spermatozoa that achieved tail elongation exhibited a dysmorphic head and a defective annulus [25]. Although both SEPT12 and Hook1 interact with tubulins during spermiogenesis, the azh strains still displayed distinct phenotypes (e.g., coiled tail and decapitation) compared with those of *SEPTIN12* chimeric mice [45,46]. We proposed a working model of the SEPT12 and α- and β-tubulins in spermiogenesis (Figure 6), in which SEPT12 is involved in sperm head formation and tail elongation. A reduced SEPT12 expression level disrupts sperm head shaping and tail formation by affecting the organization of α- and β-tubulins. In certain cells, reduced SEPT12 causes sperm head formation and tail elongation to be disturbed. Although certain cells achieve spermiogenesis, they produce a defective annulus. Our finding suggests that SEPT12 and α- and β-tubulin complexes play crucial roles in sperm head shaping and sperm tail elongation during spermiogenesis.

## Supplementary Information
