# Supplementary material for: SEPT12-Microtubule Complexes Are Required for Sperm Head and Tail Formation
Source: Int J Mol Sci. 2013 Nov 7;14(11):22102–16. doi: 10.3390/ijms141122102 (PMC3856054; doi:10.3390/ijms141122102)
Supplement: Supplementary file 1 [file ijms-14-22102-s001.pdf]

# Supplementary Information

**Figure S1.** SEPT12-GFP interacts with α-tubulin and β-tubulin in 293T cell line. Co-IP of α-tubulin (**A**) and β-tubulin (**B**) with SEPT12-GFP with. Lysates from transfected 293T cells were immunoprecipitated (IP) with anti-α-tubulin antibody (**A**. left panel, lane3), anti-β-tubulin (**B**. right panel, lane 3), or a nonspecific control IgG (left and right panel, lane 2), followed by immuno-blotting (IB) with anti-GFP antibody. Input protein (5%) was used as control of immuno-blotting (IB) in the transfected cell lysates.

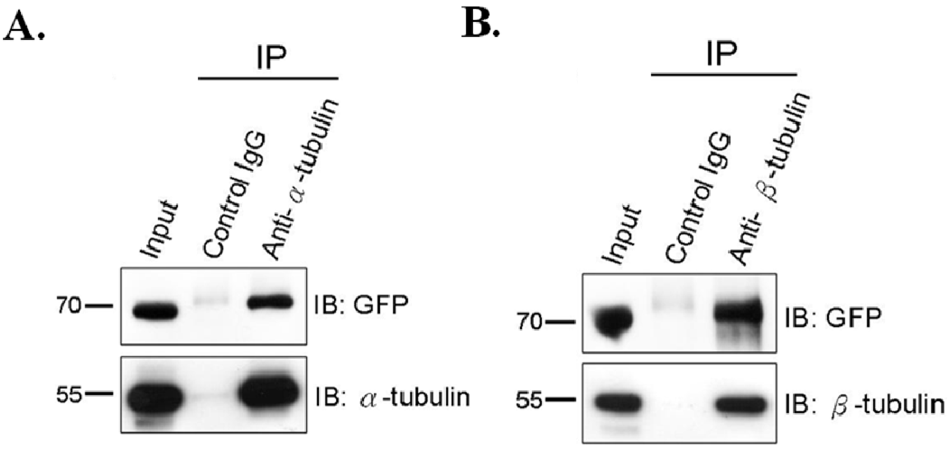


**Figure S2.** The SEPTIN12-EGFP copy number in the transgenic mice. (**A**) Real-time PCR was used to determine the SEPTIN12-EGFP copy number in the transgenic mice; (**B**) Standard curves of ectopic SEPTIN12-EGFP and endogenous Septin12; (**C**) Calculation formula of SEPTIN12-EGFP copy number.

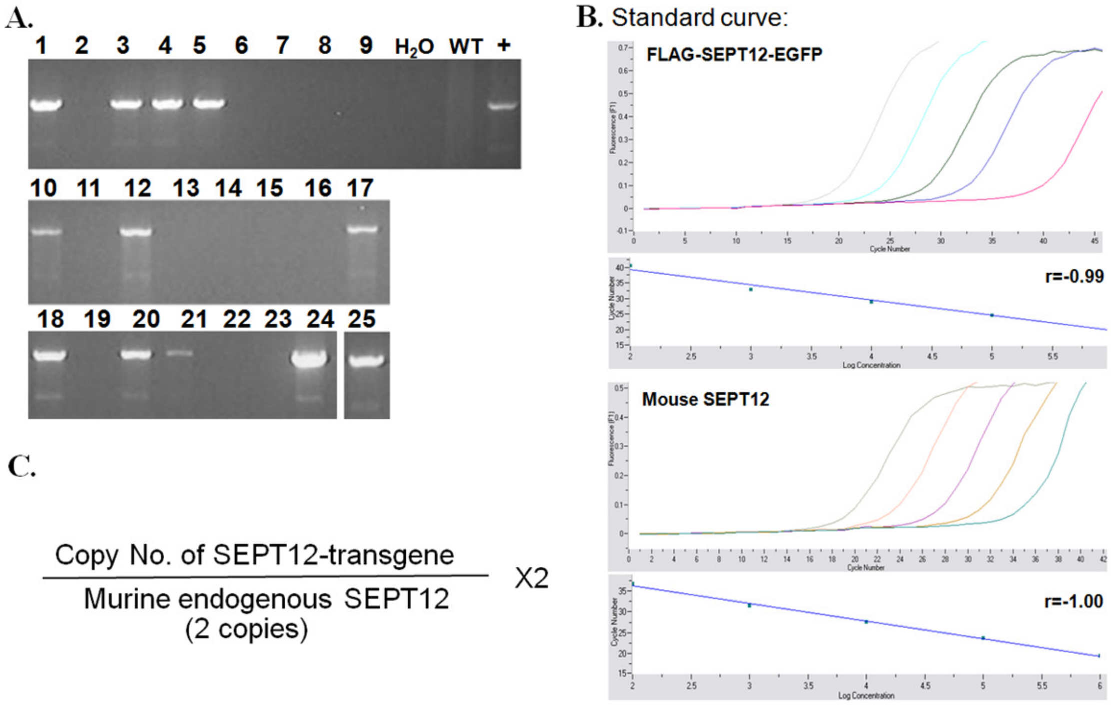

**Figure S3.** SEPT12 is co-localized with α-tubulin and β-tubulin at elongation stage of spermatids in the wild-type mice. (**A** and **B**) Immuno-fluorescence detection of SEPT12 and α-tubulin (**A**) or β-tubulin (**B**). (**a.**) early elongation stage and (**b.**) elongation stage. From left to right: DAPI (Blue), tubulin (Red), SEPT12 (green) and merged pictures for DAPI, tubulin and SEPT12.

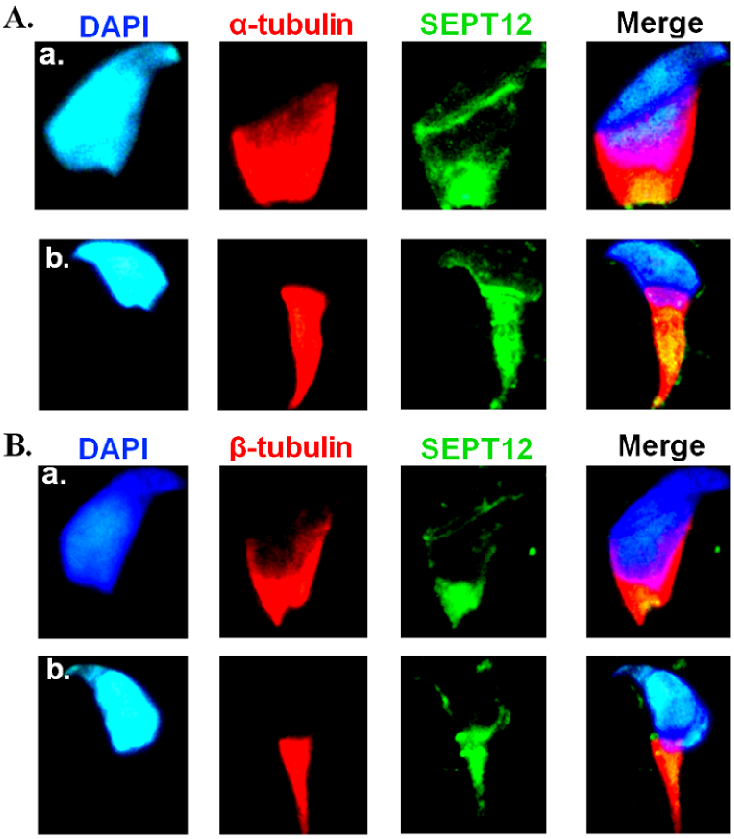


© 2013 by the authors; licensee MDPI, Basel, Switzerland. This article is an open access article distributed under the terms and conditions of the Creative Commons Attribution license (http://creativecommons.org/licenses/by/3.0/).
